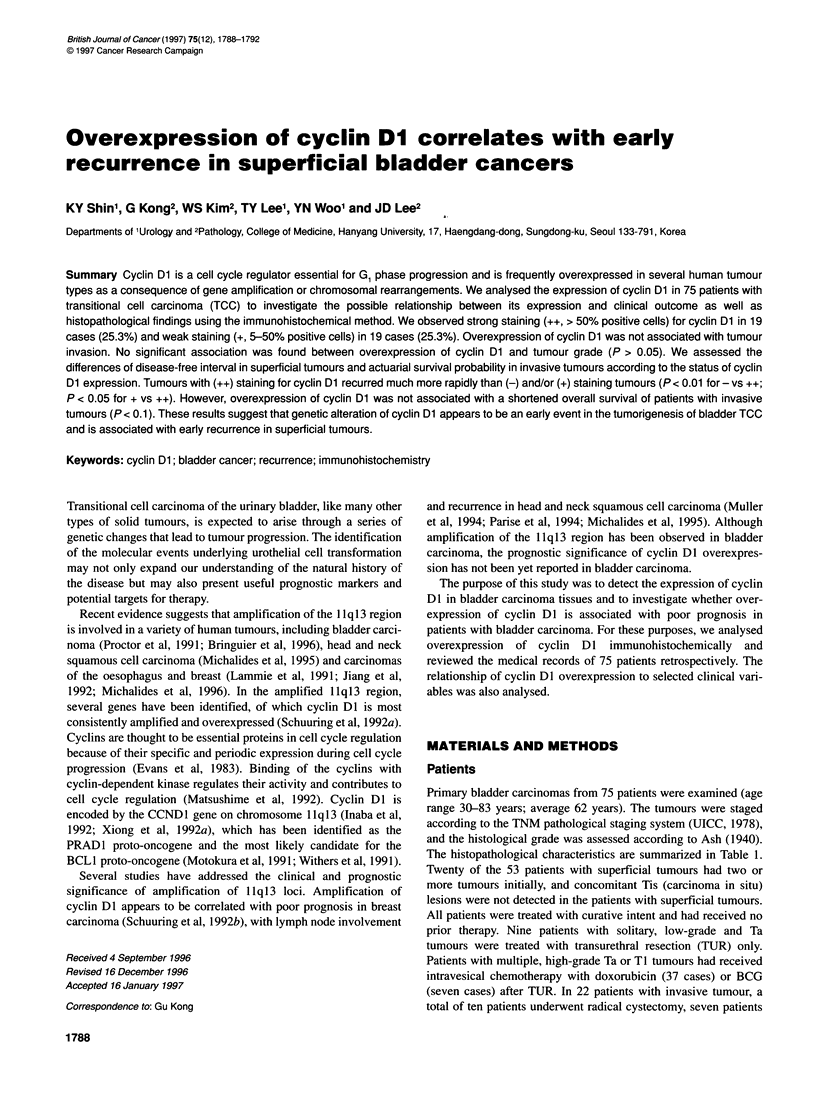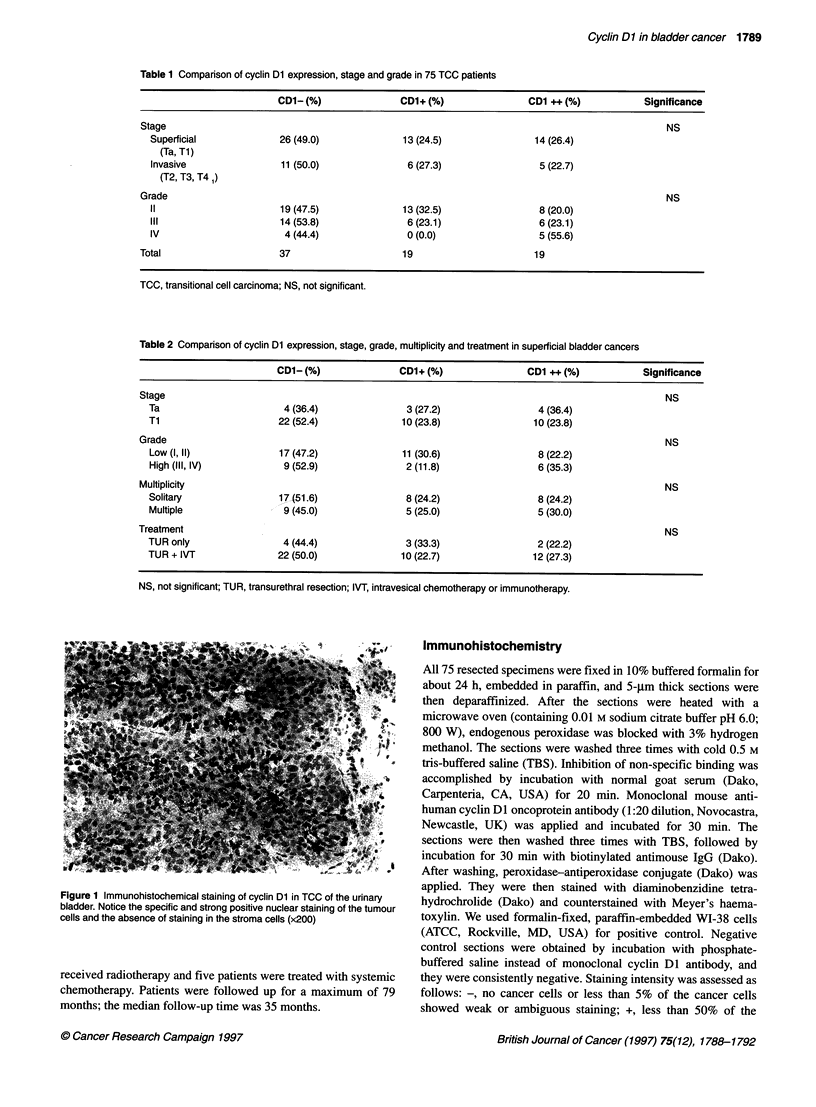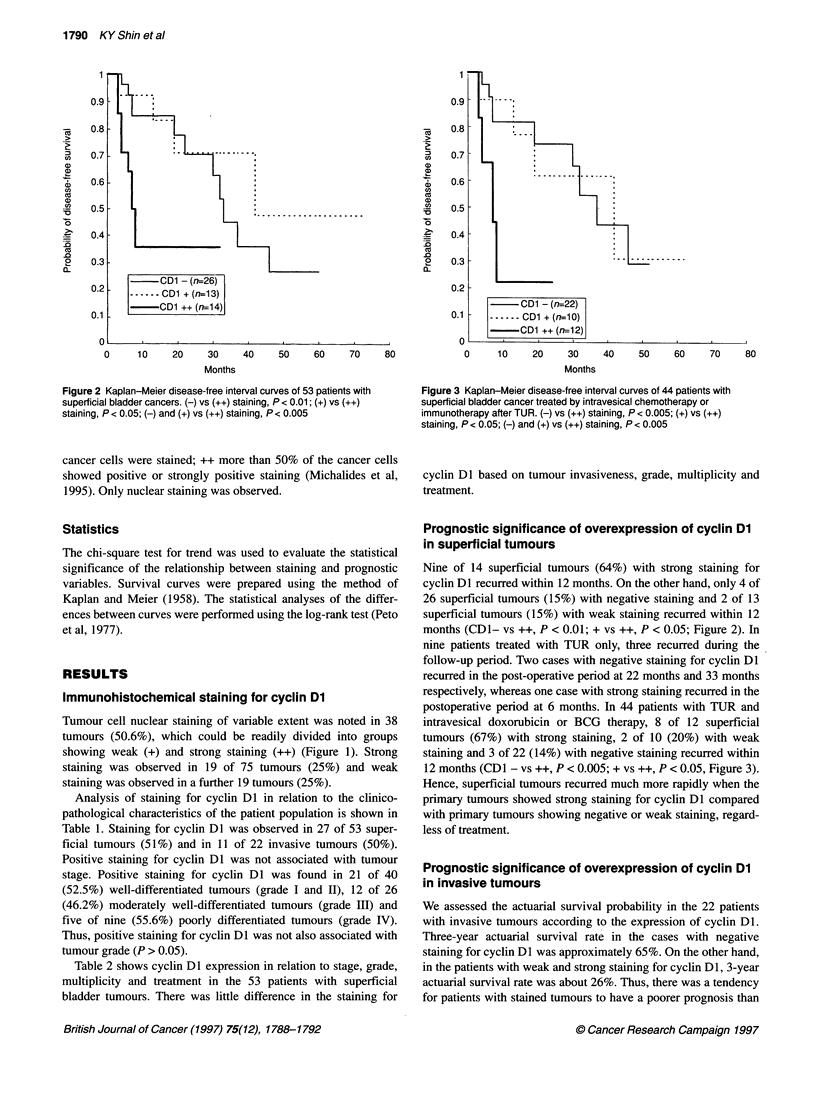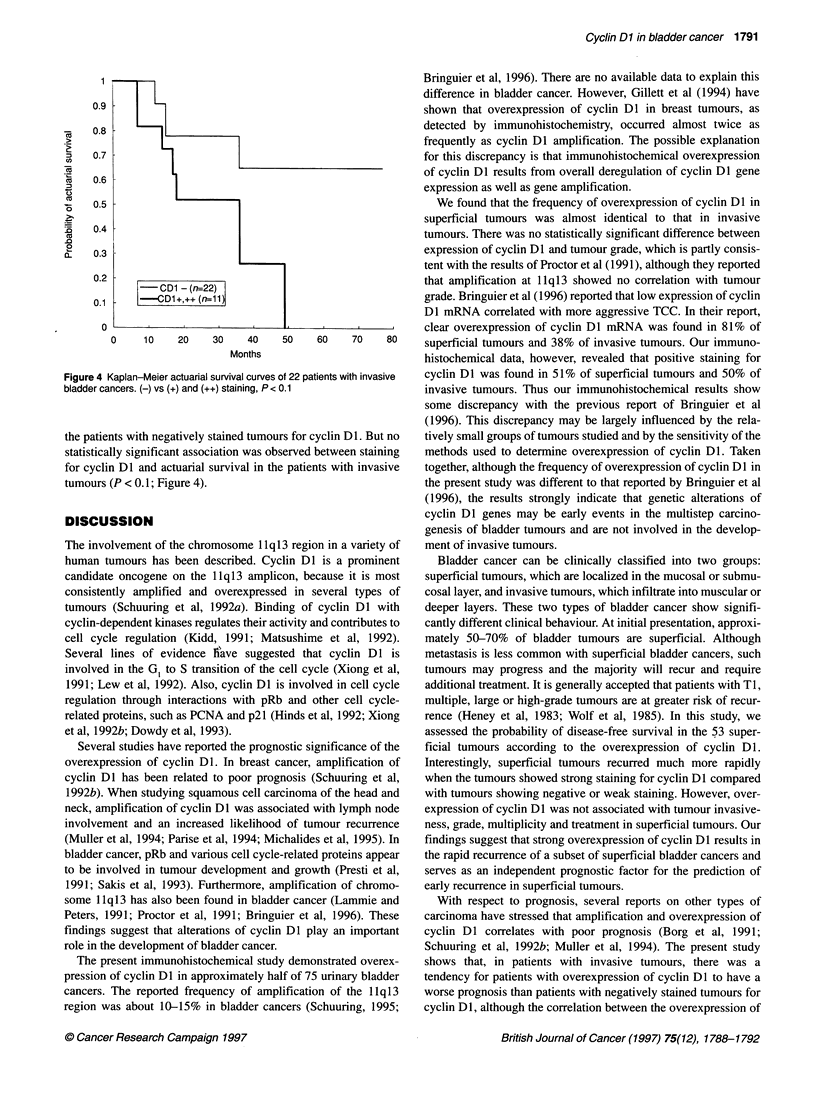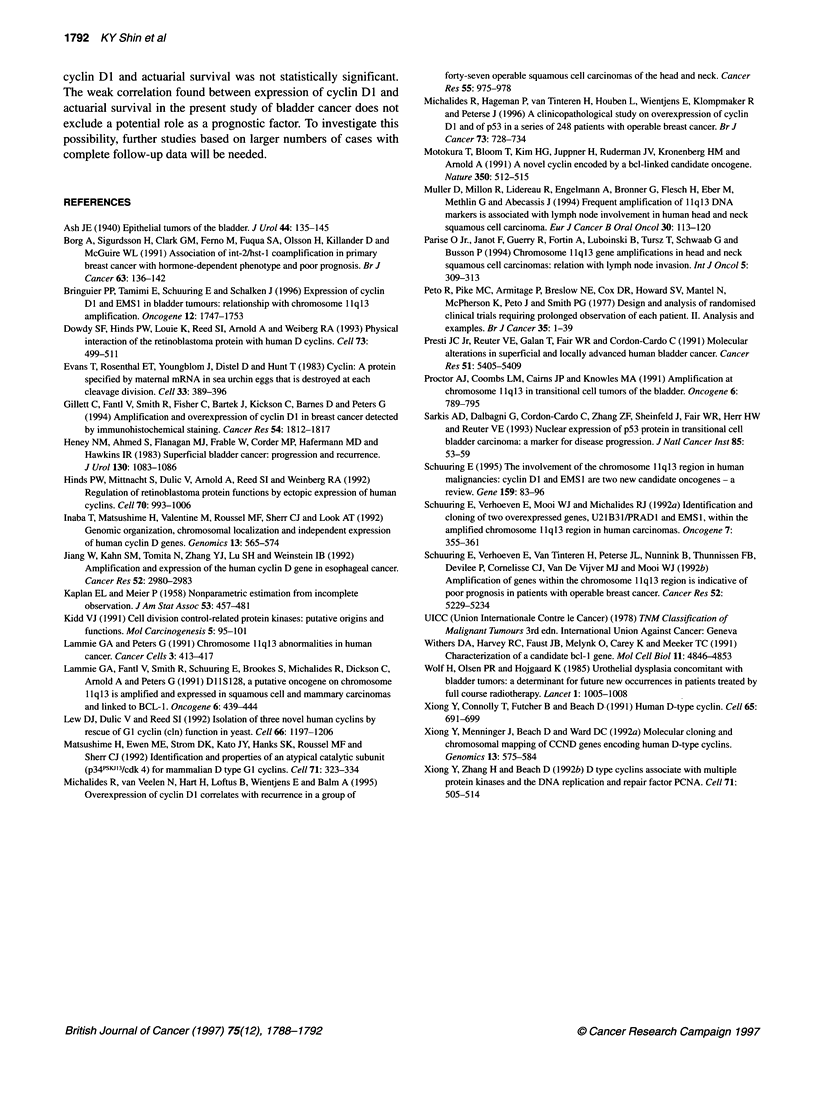# Overexpression of cyclin D1 correlates with early recurrence in superficial bladder cancers.

**DOI:** 10.1038/bjc.1997.305

**Published:** 1997

**Authors:** K. Y. Shin, G. Kong, W. S. Kim, T. Y. Lee, Y. N. Woo, J. D. Lee

**Affiliations:** Department of Urology, College of Medicine, Hanyang University, Seoul, Korea.

## Abstract

**Images:**


British Journal of Cancer (1997) 75(12), 1788–1792
© 1997 Cancer Research Campaign

### Overexpression of cyclin D1 correlates with early recurrence in superficial bladder cancers

**KY Shin[1], G Kong[2], WS Kim[2], TY Lee[1], YN Woo[1] and JD Lee[2]**

Departments of [1]Urology and [2]Pathology, College of Medicine, Hanyang University, 17, Haengdang-dong, Sungdong-ku, Seoul 133-791, Korea

**Summary** Cyclin D1 is a cell cycle regulator essential for $G_1$ phase progression and is frequently overexpressed in several human tumour types as a consequence of gene amplification or chromosomal rearrangements. We analysed the expression of cyclin D1 in 75 patients with transitional cell carcinoma (TCC) to investigate the possible relationship between its expression and clinical outcome as well as histopathological findings using the immunohistochemical method. We observed strong staining (++, > 50% positive cells) for cyclin D1 in 19 cases (25.3%) and weak staining (+, 5–50% positive cells) in 19 cases (25.3%). Overexpression of cyclin D1 was not associated with tumour invasion. No significant association was found between overexpression of cyclin D1 and tumour grade ($P > 0.05$). We assessed the differences of disease-free interval in superficial tumours and actuarial survival probability in invasive tumours according to the status of cyclin D1 expression. Tumours with (++) staining for cyclin D1 recurred much more rapidly than (–) and/or (+) staining tumours ($P < 0.01$ for – vs ++; $P < 0.05$ for + vs ++). However, overexpression of cyclin D1 was not associated with a shortened overall survival of patients with invasive tumours ($P < 0.1$). These results suggest that genetic alteration of cyclin D1 appears to be an early event in the tumorigenesis of bladder TCC and is associated with early recurrence in superficial tumours.

**Keywords:** cyclin D1; bladder cancer; recurrence; immunohistochemistry

Transitional cell carcinoma of the urinary bladder, like many other types of solid tumours, is expected to arise through a series of genetic changes that lead to tumour progression. The identification of the molecular events underlying urothelial cell transformation may not only expand our understanding of the natural history of the disease but may also present useful prognostic markers and potential targets for therapy.

Recent evidence suggests that amplification of the 11q13 region is involved in a variety of human tumours, including bladder carcinoma (Proctor et al, 1991; Bringuier et al, 1996), head and neck squamous cell carcinoma (Michalides et al, 1995) and carcinomas of the oesophagus and breast (Lammie et al, 1991; Jiang et al, 1992; Michalides et al, 1996). In the amplified 11q13 region, several genes have been identified, of which cyclin D1 is most consistently amplified and overexpressed (Schuuring et al, 1992*a*). Cyclins are thought to be essential proteins in cell cycle regulation because of their specific and periodic expression during cell cycle progression (Evans et al, 1983). Binding of the cyclins with cyclin-dependent kinase regulates their activity and contributes to cell cycle regulation (Matsushime et al, 1992). Cyclin D1 is encoded by the CCND1 gene on chromosome 11q13 (Inaba et al, 1992; Xiong et al, 1992*a*), which has been identified as the PRAD1 proto-oncogene and the most likely candidate for the BCL1 proto-oncogene (Motokura et al, 1991; Withers et al, 1991).

Several studies have addressed the clinical and prognostic significance of amplification of 11q13 loci. Amplification of cyclin D1 appears to be correlated with poor prognosis in breast carcinoma (Schuuring et al, 1992*b*), with lymph node involvement and recurrence in head and neck squamous cell carcinoma (Muller et al, 1994; Parise et al, 1994; Michalides et al, 1995). Although amplification of the 11q13 region has been observed in bladder carcinoma, the prognostic significance of cyclin D1 overexpression has not been yet reported in bladder carcinoma.

The purpose of this study was to detect the expression of cyclin D1 in bladder carcinoma tissues and to investigate whether overexpression of cyclin D1 is associated with poor prognosis in patients with bladder carcinoma. For these purposes, we analysed overexpression of cyclin D1 immunohistochemically and reviewed the medical records of 75 patients retrospectively. The relationship of cyclin D1 overexpression to selected clinical variables was also analysed.

*Received 4 September 1996*
*Revised 16 December 1996*
*Accepted 16 January 1997*

*Correspondence to*: Gu Kong

### MATERIALS AND METHODS

### Patients

Primary bladder carcinomas from 75 patients were examined (age range 30–83 years; average 62 years). The tumours were staged according to the TNM pathological staging system (UICC, 1978), and the histological grade was assessed according to Ash (1940). The histopathological characteristics are summarized in Table 1. Twenty of the 53 patients with superficial tumours had two or more tumours initially, and concomitant Tis (carcinoma in situ) lesions were not detected in the patients with superficial tumours. All patients were treated with curative intent and had received no prior therapy. Nine patients with solitary, low-grade and Ta tumours were treated with transurethral resection (TUR) only. Patients with multiple, high-grade Ta or T1 tumours had received intravesical chemotherapy with doxorubicin (37 cases) or BCG (seven cases) after TUR. In 22 patients with invasive tumour, a total of ten patients underwent radical cystectomy, seven patients

**Table 1** Comparison of cyclin D1 expression, stage and grade in 75 TCC patients

| | CD1– (%) | CD1+ (%) | CD1 ++ (%) | Significance |
|---|---|---|---|---|
| Stage | | | | NS |
| Superficial (Ta, T1) | 26 (49.0) | 13 (24.5) | 14 (26.4) | |
| Invasive (T2, T3, T4 ,) | 11 (50.0) | 6 (27.3) | 5 (22.7) | |
| Grade | | | | NS |
| II | 19 (47.5) | 13 (32.5) | 8 (20.0) | |
| III | 14 (53.8) | 6 (23.1) | 6 (23.1) | |
| IV | 4 (44.4) | 0 (0.0) | 5 (55.6) | |
| Total | 37 | 19 | 19 | |

TCC, transitional cell carcinoma; NS, not significant.

**Table 2** Comparison of cyclin D1 expression, stage, grade, multiplicity and treatment in superficial bladder cancers

| | CD1– (%) | CD1+ (%) | CD1 ++ (%) | Significance |
|---|---|---|---|---|
| Stage | | | | NS |
| Ta | 4 (36.4) | 3 (27.2) | 4 (36.4) | |
| T1 | 22 (52.4) | 10 (23.8) | 10 (23.8) | |
| Grade | | | | NS |
| Low (I, II) | 17 (47.2) | 11 (30.6) | 8 (22.2) | |
| High (III, IV) | 9 (52.9) | 2 (11.8) | 6 (35.3) | |
| Multiplicity | | | | NS |
| Solitary | 17 (51.6) | 8 (24.2) | 8 (24.2) | |
| Multiple | 9 (45.0) | 5 (25.0) | 5 (30.0) | |
| Treatment | | | | NS |
| TUR only | 4 (44.4) | 3 (33.3) | 2 (22.2) | |
| TUR + IVT | 22 (50.0) | 10 (22.7) | 12 (27.3) | |

NS, not significant; TUR, transurethral resection; IVT, intravesical chemotherapy or immunotherapy.

**Figure 1** Immunohistochemical staining of cyclin D1 in TCC of the urinary bladder. Notice the specific and strong positive nuclear staining of the tumour cells and the absence of staining in the stroma cells (×200)

received radiotherapy and five patients were treated with systemic chemotherapy. Patients were followed up for a maximum of 79 months; the median follow-up time was 35 months.

### Immunohistochemistry

All 75 resected specimens were fixed in 10% buffered formalin for about 24 h, embedded in paraffin, and 5-μm thick sections were then deparaffinized. After the sections were heated with a microwave oven (containing 0.01 M sodium citrate buffer pH 6.0; 800 W), endogenous peroxidase was blocked with 3% hydrogen methanol. The sections were washed three times with cold 0.5 M tris-buffered saline (TBS). Inhibition of non-specific binding was accomplished by incubation with normal goat serum (Dako, Carpenteria, CA, USA) for 20 min. Monoclonal mouse anti-human cyclin D1 oncoprotein antibody (1:20 dilution, Novocastra, Newcastle, UK) was applied and incubated for 30 min. The sections were then washed three times with TBS, followed by incubation for 30 min with biotinylated antimouse IgG (Dako). After washing, peroxidase–antiperoxidase conjugate (Dako) was applied. They were then stained with diaminobenzidine tetrahydrochrolide (Dako) and counterstained with Meyer's haematoxylin. We used formalin-fixed, paraffin-embedded WI-38 cells (ATCC, Rockville, MD, USA) for positive control. Negative control sections were obtained by incubation with phosphate-buffered saline instead of monoclonal cyclin D1 antibody, and they were consistently negative. Staining intensity was assessed as follows: –, no cancer cells or less than 5% of the cancer cells showed weak or ambiguous staining; +, less than 50% of the

© Cancer Research Campaign 1997

**Figure 2** Kaplan–Meier disease-free interval curves of 53 patients with superficial bladder cancers. (–) vs (++) staining, $P < 0.01$; (+) vs (++) staining, $P < 0.05$; (–) and (+) vs (++) staining, $P < 0.005$

**Figure 3** Kaplan–Meier disease-free interval curves of 44 patients with superficial bladder cancer treated by intravesical chemotherapy or immunotherapy after TUR. (–) vs (++) staining, $P < 0.005$; (+) vs (++) staining, $P < 0.05$; (–) and (+) vs (++) staining, $P < 0.005$

cancer cells were stained; ++ more than 50% of the cancer cells showed positive or strongly positive staining (Michalides et al, 1995). Only nuclear staining was observed.

### Statistics

The chi-square test for trend was used to evaluate the statistical significance of the relationship between staining and prognostic variables. Survival curves were prepared using the method of Kaplan and Meier (1958). The statistical analyses of the differences between curves were performed using the log-rank test (Peto et al, 1977).

### RESULTS

### Immunohistochemical staining for cyclin D1

Tumour cell nuclear staining of variable extent was noted in 38 tumours (50.6%), which could be readily divided into groups showing weak (+) and strong staining (++) (Figure 1). Strong staining was observed in 19 of 75 tumours (25%) and weak staining was observed in a further 19 tumours (25%).

Analysis of staining for cyclin D1 in relation to the clinicopathological characteristics of the patient population is shown in Table 1. Staining for cyclin D1 was observed in 27 of 53 superficial tumours (51%) and in 11 of 22 invasive tumours (50%). Positive staining for cyclin D1 was not associated with tumour stage. Positive staining for cyclin D1 was found in 21 of 40 (52.5%) well-differentiated tumours (grade I and II), 12 of 26 (46.2%) moderately well-differentiated tumours (grade III) and five of nine (55.6%) poorly differentiated tumours (grade IV). Thus, positive staining for cyclin D1 was not also associated with tumour grade ($P > 0.05$).

Table 2 shows cyclin D1 expression in relation to stage, grade, multiplicity and treatment in the 53 patients with superficial bladder tumours. There was little difference in the staining for cyclin D1 based on tumour invasiveness, grade, multiplicity and treatment.

### Prognostic significance of overexpression of cyclin D1 in superficial tumours

Nine of 14 superficial tumours (64%) with strong staining for cyclin D1 recurred within 12 months. On the other hand, only 4 of 26 superficial tumours (15%) with negative staining and 2 of 13 superficial tumours (15%) with weak staining recurred within 12 months (CD1– vs ++, $P < 0.01$; + vs ++, $P < 0.05$; Figure 2). In nine patients treated with TUR only, three recurred during the follow-up period. Two cases with negative staining for cyclin D1 recurred in the post-operative period at 22 months and 33 months respectively, whereas one case with strong staining recurred in the postoperative period at 6 months. In 44 patients with TUR and intravesical doxorubicin or BCG therapy, 8 of 12 superficial tumours (67%) with strong staining, 2 of 10 (20%) with weak staining and 3 of 22 (14%) with negative staining recurred within 12 months (CD1 – vs ++, $P < 0.005$; + vs ++, $P < 0.05$, Figure 3). Hence, superficial tumours recurred much more rapidly when the primary tumours showed strong staining for cyclin D1 compared with primary tumours showing negative or weak staining, regardless of treatment.

### Prognostic significance of overexpression of cyclin D1 in invasive tumours

We assessed the actuarial survival probability in the 22 patients with invasive tumours according to the expression of cyclin D1. Three-year actuarial survival rate in the cases with negative staining for cyclin D1 was approximately 65%. On the other hand, in the patients with weak and strong staining for cyclin D1, 3-year actuarial survival rate was about 26%. Thus, there was a tendency for patients with stained tumours to have a poorer prognosis than

© Cancer Research Campaign 1997

**Figure 4** Kaplan–Meier actuarial survival curves of 22 patients with invasive bladder cancers. (–) vs (+) and (++) staining, $P < 0.1$

the patients with negatively stained tumours for cyclin D1. But no statistically significant association was observed between staining for cyclin D1 and actuarial survival in the patients with invasive tumours ($P < 0.1$; Figure 4).

### DISCUSSION

The involvement of the chromosome 11q13 region in a variety of human tumours has been described. Cyclin D1 is a prominent candidate oncogene on the 11q13 amplicon, because it is most consistently amplified and overexpressed in several types of tumours (Schuuring et al, 1992*a*). Binding of cyclin D1 with cyclin-dependent kinases regulates their activity and contributes to cell cycle regulation (Kidd, 1991; Matsushime et al, 1992). Several lines of evidence have suggested that cyclin D1 is involved in the $G_1$ to S transition of the cell cycle (Xiong et al, 1991; Lew et al, 1992). Also, cyclin D1 is involved in cell cycle regulation through interactions with pRb and other cell cycle-related proteins, such as PCNA and p21 (Hinds et al, 1992; Xiong et al, 1992*b*; Dowdy et al, 1993).

Several studies have reported the prognostic significance of the overexpression of cyclin D1. In breast cancer, amplification of cyclin D1 has been related to poor prognosis (Schuuring et al, 1992*b*). When studying squamous cell carcinoma of the head and neck, amplification of cyclin D1 was associated with lymph node involvement and an increased likelihood of tumour recurrence (Muller et al, 1994; Parise et al, 1994; Michalides et al, 1995). In bladder cancer, pRb and various cell cycle-related proteins appear to be involved in tumour development and growth (Presti et al, 1991; Sakis et al, 1993). Furthermore, amplification of chromosome 11q13 has also been found in bladder cancer (Lammie and Peters, 1991; Proctor et al, 1991; Bringuier et al, 1996). These findings suggest that alterations of cyclin D1 play an important role in the development of bladder cancer.

The present immunohistochemical study demonstrated overexpression of cyclin D1 in approximately half of 75 urinary bladder cancers. The reported frequency of amplification of the 11q13 region was about 10–15% in bladder cancers (Schuuring, 1995; Bringuier et al, 1996). There are no available data to explain this difference in bladder cancer. However, Gillett et al (1994) have shown that overexpression of cyclin D1 in breast tumours, as detected by immunohistochemistry, occurred almost twice as frequently as cyclin D1 amplification. The possible explanation for this discrepancy is that immunohistochemical overexpression of cyclin D1 results from overall deregulation of cyclin D1 gene expression as well as gene amplification.

We found that the frequency of overexpression of cyclin D1 in superficial tumours was almost identical to that in invasive tumours. There was no statistically significant difference between expression of cyclin D1 and tumour grade, which is partly consistent with the results of Proctor et al (1991), although they reported that amplification at 11q13 showed no correlation with tumour grade. Bringuier et al (1996) reported that low expression of cyclin D1 mRNA correlated with more aggressive TCC. In their report, clear overexpression of cyclin D1 mRNA was found in 81% of superficial tumours and 38% of invasive tumours. Our immunohistochemical data, however, revealed that positive staining for cyclin D1 was found in 51% of superficial tumours and 50% of invasive tumours. Thus our immunohistochemical results show some discrepancy with the previous report of Bringuier et al (1996). This discrepancy may be largely influenced by the relatively small groups of tumours studied and by the sensitivity of the methods used to determine overexpression of cyclin D1. Taken together, although the frequency of overexpression of cyclin D1 in the present study was different to that reported by Bringuier et al (1996), the results strongly indicate that genetic alterations of cyclin D1 genes may be early events in the multistep carcinogenesis of bladder tumours and are not involved in the development of invasive tumours.

Bladder cancer can be clinically classified into two groups: superficial tumours, which are localized in the mucosal or submucosal layer, and invasive tumours, which infiltrate into muscular or deeper layers. These two types of bladder cancer show significantly different clinical behaviour. At initial presentation, approximately 50–70% of bladder tumours are superficial. Although metastasis is less common with superficial bladder cancers, such tumours may progress and the majority will recur and require additional treatment. It is generally accepted that patients with T1, multiple, large or high-grade tumours are at greater risk of recurrence (Heney et al, 1983; Wolf et al, 1985). In this study, we assessed the probability of disease-free survival in the 53 superficial tumours according to the overexpression of cyclin D1. Interestingly, superficial tumours recurred much more rapidly when the tumours showed strong staining for cyclin D1 compared with tumours showing negative or weak staining. However, overexpression of cyclin D1 was not associated with tumour invasiveness, grade, multiplicity and treatment in superficial tumours. Our findings suggest that strong overexpression of cyclin D1 results in the rapid recurrence of a subset of superficial bladder cancers and serves as an independent prognostic factor for the prediction of early recurrence in superficial tumours.

With respect to prognosis, several reports on other types of carcinoma have stressed that amplification and overexpression of cyclin D1 correlates with poor prognosis (Borg et al, 1991; Schuuring et al, 1992*b*; Muller et al, 1994). The present study shows that, in patients with invasive tumours, there was a tendency for patients with overexpression of cyclin D1 to have a worse prognosis than patients with negatively stained tumours for cyclin D1, although the correlation between the overexpression of

© Cancer Research Campaign 1997

cyclin D1 and actuarial survival was not statistically significant. The weak correlation found between expression of cyclin D1 and actuarial survival in the present study of bladder cancer does not exclude a potential role as a prognostic factor. To investigate this possibility, further studies based on larger numbers of cases with complete follow-up data will be needed.

### REFERENCES

Ash JE (1940) Epithelial tumors of the bladder. *J Urol* **44**: 135–145

Borg A, Sigurdsson H, Clark GM, Ferno M, Fuqua SA, Olsson H, Killander D and McGuire WL (1991) Association of int-2/hst-1 coamplification in primary breast cancer with hormone-dependent phenotype and poor prognosis. *Br J Cancer* **63**: 136–142

Bringuier PP, Tamimi E, Schuuring E and Schalken J (1996) Expression of cyclin D1 and EMS1 in bladder tumours: relationship with chromosome 11q13 amplification. *Oncogene* **12**: 1747–1753

Dowdy SF, Hinds PW, Louie K, Reed SI, Arnold A and Weiberg RA (1993) Physical interaction of the retinoblastoma protein with human D cyclins. *Cell* **73**: 499–511

Evans T, Rosenthal ET, Youngblom J, Distel D and Hunt T (1983) Cyclin: A protein specified by maternal mRNA in sea urchin eggs that is destroyed at each cleavage division. *Cell* **33**: 389–396

Gillett C, Fantl V, Smith R, Fisher C, Bartek J, Kickson C, Barnes D and Peters G (1994) Amplification and overexpression of cyclin D1 in breast cancer detected by immunohistochemical staining. *Cancer Res* **54**: 1812–1817

Heney NM, Ahmed S, Flanagan MJ, Frable W, Corder MP, Hafermann MD and Hawkins IR (1983) Superficial bladder cancer: progression and recurrence. *J Urol* **130**: 1083–1086

Hinds PW, Mittnacht S, Dulic V, Arnold A, Reed SI and Weinberg RA (1992) Regulation of retinoblastoma protein functions by ectopic expression of human cyclins. *Cell* **70**: 993–1006

Inaba T, Matsushime H, Valentine M, Roussel MF, Sherr CJ and Look AT (1992) Genomic organization, chromosomal localization and independent expression of human cyclin D genes. *Genomics* **13**: 565–574

Jiang W, Kahn SM, Tomita N, Zhang YJ, Lu SH and Weinstein IB (1992) Amplification and expression of the human cyclin D gene in esophageal cancer. *Cancer Res* **52**: 2980–2983

Kaplan EL and Meier P (1958) Nonparametric estimation from incomplete observation. *J Am Stat Assoc* **53**: 457–481

Kidd VJ (1991) Cell division control-related protein kinases: putative origins and functions. *Mol Carcinogenesis* **5**: 95–101

Lammie GA and Peters G (1991) Chromosome 11q13 abnormalities in human cancer. *Cancer Cells* **3**: 413–417

Lammie GA, Fantl V, Smith R, Schuuring E, Brookes S, Michalides R, Dickson C, Arnold A and Peters G (1991) D11S128, a putative oncogene on chromosome 11q13 is amplified and expressed in squamous cell and mammary carcinomas and linked to BCL-1. *Oncogene* **6**: 439–444

Lew DJ, Dulic V and Reed SI (1992) Isolation of three novel human cyclins by rescue of G1 cyclin (cln) function in yeast. *Cell* **66**: 1197–1206

Matsushime H, Ewen ME, Strom DK, Kato JY, Hanks SK, Roussel MF and Sherr CJ (1992) Identification and properties of an atypical catalytic subunit ($p34^{PSKJ13}$/cdk 4) for mammalian D type G1 cyclins. *Cell* **71**: 323–334

Michalides R, van Veelen N, Hart H, Loftus B, Wientjens E and Balm A (1995) Overexpression of cyclin D1 correlates with recurrence in a group of forty-seven operable squamous cell carcinomas of the head and neck. *Cancer Res* **55**: 975–978

Michalides R, Hageman P, van Tinteren H, Houben L, Wientjens E, Klompmaker R and Peterse J (1996) A clinicopathological study on overexpression of cyclin D1 and of p53 in a series of 248 patients with operable breast cancer. *Br J Cancer* **73**: 728–734

Motokura T, Bloom T, Kim HG, Juppner H, Ruderman JV, Kronenberg HM and Arnold A (1991) A novel cyclin encoded by a bcl-linked candidate oncogene. *Nature* **350**: 512–515

Muller D, Millon R, Lidereau R, Engelmann A, Bronner G, Flesch H, Eber M, Methlin G and Abecassis J (1994) Frequent amplification of 11q13 DNA markers is associated with lymph node involvement in human head and neck squamous cell carcinoma. *Eur J Cancer B Oral Oncol* **30**: 113–120

Parise O Jr., Janot F, Guerry R, Fortin A, Luboinski B, Tursz T, Schwaab G and Busson P (1994) Chromosome 11q13 gene amplifications in head and neck squamous cell carcinomas: relation with lymph node invasion. *Int J Oncol* **5**: 309–313

Peto R, Pike MC, Armitage P, Breslow NE, Cox DR, Howard SV, Mantel N, McPherson K, Peto J and Smith PG (1977) Design and analysis of randomised clinical trials requiring prolonged observation of each patient. II. Analysis and examples. *Br J Cancer* **35**: 1–39

Presti JC Jr, Reuter VE, Galan T, Fair WR and Cordon-Cardo C (1991) Molecular alterations in superficial and locally advanced human bladder cancer. *Cancer Res* **51**: 5405–5409

Proctor AJ, Coombs LM, Cairns JP and Knowles MA (1991) Amplification at chromosome 11q13 in transitional cell tumors of the bladder. *Oncogene* **6**: 789–795

Sarkis AD, Dalbagni G, Cordon-Cardo C, Zhang ZF, Sheinfeld J, Fair WR, Herr HW and Reuter VE (1993) Nuclear expression of p53 protein in transitional cell bladder carcinoma: a marker for disease progression. *J Natl Cancer Inst* **85**: 53–59

Schuuring E (1995) The involvement of the chromosome 11q13 region in human malignancies: cyclin D1 and EMS1 are two new candidate oncogenes – a review. *Gene* **159**: 83–96

Schuuring E, Verhoeven E, Mooi WJ and Michalides RJ (1992*a*) Identification and cloning of two overexpressed genes, U21B31/PRAD1 and EMS1, within the amplified chromosome 11q13 region in human carcinomas. *Oncogene* **7**: 355–361

Schuuring E, Verhoeven E, Van Tinteren H, Peterse JL, Nunnink B, Thunnissen FB, Devilee P, Cornelisse CJ, Van De Vijver MJ and Mooi WJ (1992*b*) Amplification of genes within the chromosome 11q13 region is indicative of poor prognosis in patients with operable breast cancer. *Cancer Res* **52**: 5229–5234

UICC (Union Internationale Contre le Cancer) (1978) *TNM Classification of Malignant Tumours* 3rd edn. International Union Against Cancer: Geneva

Withers DA, Harvey RC, Faust JB, Melynk O, Carey K and Meeker TC (1991) Characterization of a candidate bcl-1 gene. *Mol Cell Biol* **11**: 4846–4853

Wolf H, Olsen PR and Hojgaard K (1985) Urothelial dysplasia concomitant with bladder tumors: a determinant for future new occurrences in patients treated by full course radiotherapy. *Lancet* **1**: 1005–1008

Xiong Y, Connolly T, Futcher B and Beach D (1991) Human D-type cyclin. *Cell* **65**: 691–699

Xiong Y, Menninger J, Beach D and Ward DC (1992*a*) Molecular cloning and chromosomal mapping of CCND genes encoding human D-type cyclins. *Genomics* **13**: 575–584

Xiong Y, Zhang H and Beach D (1992*b*) D type cyclins associate with multiple protein kinases and the DNA replication and repair factor PCNA. *Cell* **71**: 505–514

© Cancer Research Campaign 1997